# Emergence of divergent enterovirus (EV) D68 sub-clade D1 strains, northern Italy, September to October 2018

**DOI:** 10.2807/1560-7917.ES.2018.24.7.1900090

**Published:** 2019-02-14

**Authors:** Laura Pellegrinelli, Federica Giardina, Giovanna Lunghi, Sara Colonia Uceda Renteria, Letizia Greco, Alice Fratini, Cristina Galli, Antonio Piralla, Sandro Binda, Elena Pariani, Fausto Baldanti

**Affiliations:** 1 Department of Biomedical Sciences for Health, University of Milan, Milan, Italy; 2 Molecular Virology Unit, Microbiology and Virology Department, Fondazione IRCCS Policlinico San Matteo, Pavia, Italy; 3 Microbiology and Virology Unit, Fondazione IRCCS Ca' Granda Ospedale Maggiore Policlinico, Milan, Italy; 4 Department of Clinical, Surgical, Diagnostic and Pediatric Sciences, University of Pavia, Pavia, Italy

**Keywords:** enterovirus D68, respiratory infection, phylogenetic analysis, molecular surveillance, EV-D68

## Abstract

Between September and October 2018, an enterovirus D68 (EV-D68) outbreak occurred in patients hospitalised with severe acute respiratory infection in northern Italy; 21 laboratory-confirmed cases were reported. Phylogenetic analysis revealed that 16/20 of the EV-D68 sequences belonged to a divergent group within the sub-clade D1. Since its upsurge, EV-D68 has undergone rapid evolution with the emergence of new viral variants, emphasising the need for molecular surveillance that include outpatients with respiratory illness.

An enterovirus D68 (EV-D68) outbreak was recognised at a major tertiary centre and research hospital in Milan and Pavia in the Lombardy region, northern Italy, respectively, between September and October 2018. Here, we present the molecular and clinical characteristics of the confirmed EV-D68 cases detected during the outbreak sustained by EV-D68 sub-clade D1 strains.

## Enterovirus D68 detection

From 1 August to 30 October 2018, routine laboratory testing on 853 respiratory samples, collected from patients hospitalised with respiratory infection, were analysed using molecular assays to detect causing respiratory viruses and bacteria. Of these, 91 (10.7%) tested positive for rhinovirus/EV and were further analysed using a real-time one-step RT (reverse-transcription)-PCR assay targeting the 5’ untranslated region (5’UTR) of EV-D68 [1,2]; 21 samples were positive for EV-D68 and no other viruses or bacteria were identified. Among EV-D68-positive samples, 10 were nasopharyngeal aspirates (NPA), eight nasopharyngeal swabs (NS) and three broncho-alveolar lavages (BAL).

## Characteristics of patients with enterovirus D68 infection

Of 21 EV-D68-positive cases, the median age was 18 years (range: 1 month–84 years) and 14 were male. An underlying or concurrent condition was reported for 10 patients (Table). Underlying conditions were: genetic/metabolic disorder (n = 4); leukaemia (n = 3); cystic fibrosis (n = 1); chronic heart disease (n = 1); and lymphoma (n = 1). Sixteen of 21 EV-D68-positive patients had signs and/or symptoms of lower respiratory tract infection (LRTI) and five showed symptoms of mild respiratory infection (Table). Among LRTI cases, five patients required intensive care unit (ICU) admission due to respiratory failure.

**Table:** Characteristics of patients with enterovirus D68 infection, northern Italy, 1 August–30 October 2018 (n = 21)

Age group (years)	Underlying disease	Respiratory illness	ICU admission	Type of sample analysed	Sample collection month	Strain name	EV-D68 sub-clade
80–89	Yes	Bronchiolitis	No	NS	September	EV-ITA-684–2018	D1-like
20–29	Yes	SARI and bronchial asthma	Yes	NPA	September	EV-ITA-685–2018	D1-like
40–49	Yes	Bronchiolitis	No	NS	September	EV-ITA-686–2018	D1-like
60–69	NA	Rhinorrhea	No	NS	September	EV-ITA-19478–2018	D1-like
1–9	No	Pneumonia	No	NS	September	EV-ITA-19704–2018	D1-like
70–79	NA	Pneumonia	No	BAL	September	EV-ITA-19705–2018	D1-like
<1	No	Recurrent bronchospasms	No	NS	September	EV-ITA-19957–2018	D1-like
70–79	Yes	Pneumonia	No	NS	September	EV-ITA-689–2018	D1-like
<1	No	Febrile wheezing	No	NPA	September	EV-ITA-20100–2018	D1-like
30–39	NA	Rhinorrhea and cough	No	NS	September	EV-ITA-20181–2018	D1-like
10–19	Yes	SARI and bronchial asthma	Yes	BAL	September	EV-ITA-687–2018	D1-like
1–9	No	SARI and bronchial asthma	Yes	NPA	September	EV-ITA-688–2018	B2-like
20–29	Yes	SARI and bronchial asthma	No	NPA	September	EV-ITA-690–2018	D1-like
10–19	No	SARI and bronchial asthma	Yes	NPA	September	EV-ITA-683–2018	D1-like
1–9	Yes	Rhinorrhea and cough	No	NPA	September	EV-ITA-693–2018	D1-like
1–9	Yes	SARI and bronchial asthma	Yes	NPA	September	EV-ITA-695–2018	B2-like
60–69	Yes	Pneumonia	No	NS	October	EV-ITA-694–2018	D1-like
1–9	No	SARI and bronchial asthma	No	NPA	October	EV-ITA-696–2018	NA
1–9	No	Rhinorrhea and cough	No	NPA	October	EV-ITA-751–2018	B2-like
<1	No	SARI and bronchial asthma	No	NPA	October	EV-ITA-753–2018	B2-like
80–89	Yes	Cough and pneumonia	No	BAL	October	EV-ITA-757–2018	D1-like

BAL: broncho-alveolar lavage; EV-D68: enterovirus D68; ICU: intensive care unit; NA: not available; NPA: nasopharyngeal aspirate; NS: nasal swab; SARI: severe acute respiratory infection.

## Phylogenetic and molecular analysis

Complete viral protein (VP)1 nt sequences [3] were obtained for 20 of 21 EV-D68 strains (GenBank accession numbers: MK301336-MK301355). A maximum likelihood phylogenetic tree was inferred using IQ-Tree web server (v1.6.8) [4] and the robustness of branches was evaluated using the Shimodaira–Hasegawa approximate likelihood-ratio test (SH-aLRT) and ultrafast bootstrap approximation tests.

Unexpectedly, most (16/20) sequences clustered into a distinct genetic group within the D clade, with a bootstrap value of 99% (Figure). Clade D has recently been reclassified into two sub-clades (referred as D1 and D2) [5,6]. The Italian strains clustered with one Taiwanese strain collected in 2016, one United States (US) strain collected in 2017 and 13 French strains collected in 2018 (shown in green in Figure), which together composed a divergent D1-like group. The average nt identity between EV-D68 strains belonging to this divergent group was 98.0%; the sequences shared an average nt identity of 96.3% (range: 94.7–97.8%) with all available D1 strains (n = 69) retrieved from GenBank and 93.2% (range: 92.1–94.4%) with all available D2 strains (n = 7). The remaining four EV-D68 strains detected during the outbreak belonged to sub-clade B2 and clustered with strains collected in 2018 in France.

**Figure:**
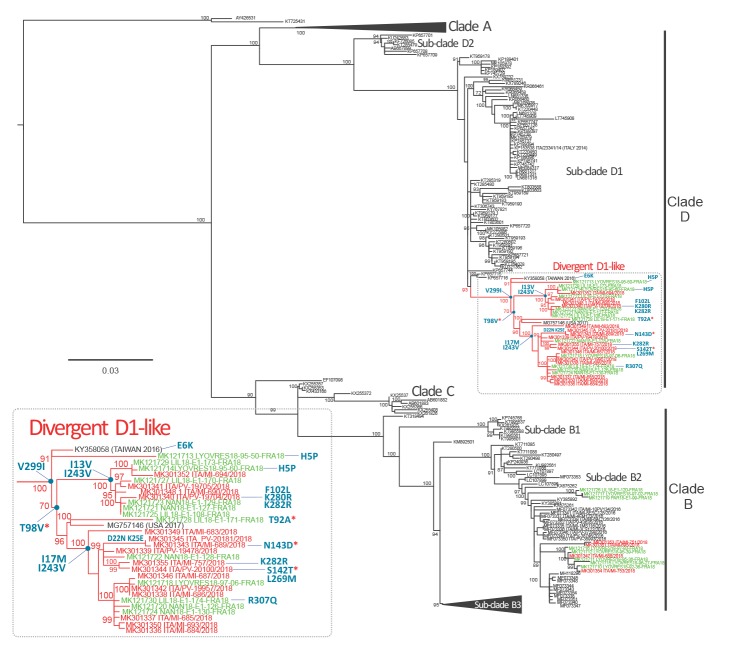
Phylogenetic rooted tree inferred by maximum likelihood of complete enterovirus D68 VP1 sequences, northern Italy, 1 August–30 October 2018 (n = 20)

EV-D68: enterovirus D68; VP: viral protein.

A Tamura 3-parameter substitution model with a gamma distribution among-site rate variation was selected as the best-fit model by ModelTest in MEGA software 7.0.21 [21]. Significant branch support values (>70%) are given for maximum-likelihood implementations (IQ-TREE bootstrap support) and are reported near the branch nodes. The EV-D68 strains identified in this study are reported in red. EV-D68 strains identified in France in 2018 [22] are reported in green. Amino acids residues characterising divergent D1-like strains are indicated in blue, including mutations in the BC and DE loops reported with an asterisk.

All VP1 sequences of EV-D68 strains belonging to the divergent D1-like group differed from sub-clade D1 by the V299I change. A series of polymorphisms, such as T98V and I243V, was observed in several subgroups of sequences. In addition, 12 of 16 Italian sequences harboured the I17M change and the remaining four strains showed the I13V change. Four polymorphisms (98V, 102L, 142T and 143D) observed in the Italian strains were within codons of protein motifs corresponding to the BC and DE loops (Figure).

## Discussion and conclusion

In 2008, EV-D68 was first reported in Italy [3,7]. In 2014 and 2016, the US and several European countries experienced a surge in the number of severe acute respiratory infection cases caused by EV-D68 infection, sustained by strains belonging to multiple EV-D68 clades [8,9];

These outbreaks, in particular the one that occurred in 2016, were chronologically and geographically superimposed by an unprecedented cluster of acute flaccid myelitis (AFM) cases, with a striking clinical similarity to poliomyelitis in patients with respiratory prodromal illness [8]. Two AFM cases associated with EV-D68 were recognised in the same period in Italy [10,11]. The association between EV-D68 infection and AFM was confirmed recently [12-14], triggering public health concern [15]. Following the 2014 outbreak, improved EV-D68 surveillance has revealed EV-D68 circulation in several countries in Europe and North America [2,8,16].

In our study, none of EV-D68-positive patients showed signs or symptoms of neurological impairment. However, in line with previous reports [17-19], 16 of 21 EV-D68-positive patients in our series had severe respiratory infections and five of them required ICU admission. A limitation of our series is that we included only inpatients with clinical illness that required hospitalisation, thus the severity of EV-D68 infections may have been overestimated. In addition, presence of underlying conditions in some patients could have made the outcome of EV-D68 infection more severe. Involving general practitioners in EV-D68 surveillance among outpatients with respiratory illness could help improve the current knowledge regarding the clinical characteristics of EV-D68 infection and the proportion of severe disease among those infected.

The phylogenetic analysis of EV*-*D68 sequences indicates that, over the past two decades, four clades (A to D) and multiple sub-lineages have emerged and spread rapidly worldwide [5,8,16]. The phylogenetic analysis of EV-D68 strains identified during the outbreak in Italy and in France (August–November 2018), underlined a wave of EV-D68 activity in Europe and revealed the presence of EV-D68 strains segregating into a divergent group within sub-clade D1 sequences. Based on the genetic distance analyses, this group of new D1-like sequences showed less genetic divergence from D1 (average 3.7%) than that previously observed between D1 and D2 (average 5.2%) [6]; we thus cannot consider this group as a new sub-clade. The re-emergence of EV-D68 strains belonging to two ‘old’ sub-clades (B2 and D1) suggests that amino acid variations have occurred over the last few years. Several amino acidic changes were observed in residues located on the surface BC and DE loops resulting in a diversification of antigenic epitopes [20]. These polymorphisms may be signatures of these divergent D1 strains, it is unclear, however, whether they possess a selective advantage. Overall, a continuous emergence and replacement of clades and sub-clades with a biennial pattern of circulation has been observed worldwide [5,8].

Our findings raise attention of the rapid emergence of new EV-D68 variants that could be associated with unexpected and possible severe clinical syndromes such as AFM. So far there is no direct evidence of the association of neurological impairment with a specific EV-D68 variant. Studies are needed to investigate the host-pathogen relationship during infection and to clarify the association between genetic diversity and clinical outcome. Sharing virological, epidemiological and clinical data on EV-D68 will enable a better understanding of the elusive pathogenesis of EV-D68.
